# Altered Skin Permeation of Finasteride Using Clove Oil, Urea, and Lyophilized Powder of Grape Seed Extract

**DOI:** 10.34172/apb.2023.010

**Published:** 2022-01-05

**Authors:** Anayatollah Salimi, Hamid Mohammad Soleymani, Saeed Mohammad Soleymani

**Affiliations:** ^1^Nanotechnology Research Center, Ahvaz Jundishapur University of Medical Sciences, Ahvaz, IR Iran.; ^2^Department of Pharmaceutics, School of Pharmacy, Ahvaz Jundishapur University of Medical Sciences, Ahvaz, IR Iran.; ^3^Petroleum Research Laboratory, School of Chemical Engineering, Iran University of Science and Technology, Tehran, Iran.; ^4^Department of Clinical Pharmacy, School of Pharmacy, Shahid Beheshti University of Medical Sciences, Tehran, Iran.

**Keywords:** Differential scanning calorimetry, Clove oil, Dermal delivery, Finasteride, FTIR, Permeability enhancers

## Abstract

***Purpose:*** Finasteride is a 5-alpha reductase inhibitor used to treat hair loss and acne. The skin permeation of finasteride is one of the main challenges associated with dermal drug delivery. One way to overcome the skin barrier is to use penetration enhancers. The purpose of this study was to investigate the effect of some penetration enhancers on finasteride permeability on the skin, as well as the effect of pretreatment time on their efficacy.

***Methods:*** In order to determine the effect of penetration enhancers on the skin permeability of finasteride, the skin was exposed to clove oil, urea, and lyophilized powder of grape seed extract (LPGSE) at different pretreatment times (2, 4 h), and then the permeability parameters were determined by passing the drug through the skin.

***Results:*** The results of this study showed that clove oil, urea, and LPGSE increased the transfer of finasteride from the skin. The highest rate of permeation was observed with clove oil (4 h), and the least permeability was observed with urea (4 h).

***Conclusion:*** Increasing the pretreatment time with clove oil and LPGSE increases the permeability of finasteride. Meanwhile, the increase in pretreatment time with urea reduces the penetration of finasteride from the skin due to reversible effects.

## Introduction

 5α-Reductase is an enzyme produced by the skin and other body organs. This enzyme increases male traits in people by converting testosterone to dihydrotestosterone (DHT).^1^ DHT produced in the skin, in addition to its physiologic effects, can lead to acne or hair loss.^2^

 finasteride is an enzyme inhibitor that inhibits the conversion of testosterone into DHT by inhibiting 5-alpha reductase.^3^ It has good gastrointestinal absorption and high bioavailability (65%).^4,5^ The oral or systemic administration of finasteride has many side effects.^6^ Therefore, according to the DHT function in the tissue, the use of topical products is recommended.^7^ In fact, the dermal use of finasteride can be considered a targeted drug delivery method that eliminates systemic adverse effects.^8^ finasteride has a molecular weight of 372.5 Da and low solubility in water (11.7 mg/L, log p 3). Its low molecular weight makes it a candidate for the delivery of drugs via the skin.^9-11^

 Two medication administration routes are transdermal and dermal.^12^ The main advantages of these systems are related to the elimination of the primary liver metabolism, contact with biological conditions and gastrointestinal chemicals, undesirable events, and the ability to provide a controlled drug delivery system for medications with a short half-life and a narrow therapeutic index.^13^

 The possibility of topical administration depends on the permeability of the drug and its therapeutic concentration.^14^ Although the nature of the skin prevents the penetration of many drugs, and drug delivery in such a way is often difficult, but it has several benefits for drug delivery.^15^ The main obstacle preventing dermal delivery is the stratum corneum.^16^

 One of the approaches for increasing the absorption of drugs through the skin is the use of penetration enhancers. Many compounds have been used for this purpose, including sulfoxides, azones, pyrrolidones, alcohols and alkanols, glycols, surfactants, and terpenes.^17^ Previous studies have shown that natural products can be used to increase the penetration of drugs through the skin. Some studies have shown that these materials are even more permeable than other penetration enhancers. Furthermore, they are safe, non-toxic, pharmaceutically inert, non-irritant, and non-allergic.^18^

 Clove oil is an essential oil that is used as a penetration enhancer.^19^ Urea and its derivatives are among the most-used and oldest permeability enhancers.^20^ Although lyophilized powder of grape seed extract (LPGSE) has been acknowledged as a polyphenol in dermatological products, no research has been conducted on the effect of LPGSE powder on penetration enhancement.^21^

 This study aims to assess the impact of various factors on finasteride’s influence on skin permeability. Also, in this study, the effect of skin penetration enhancers’ exposure times on the skin permeability of finasteride has been investigated.

## Materials and Methods

###  Materials

 Finasteride was purchased from a pharmaceutical company (Soha Helal, Tehran, Iran). Urea and clove oil were obtained from the Barij Essence Iranian Company (Kashan, Iran). LPGSE was gifted from Roshd Center (Ahvaz Jundishapur University of Medical Sciences).

###  Animal experiments

 Male rats (200-250 g; 8-10 weeks old) were provided by the Animal Laboratory of the Ahvaz Jundishapur University of Medical Sciences. All animals were anesthetized before being sacrificed with high concentrations of thiopental sodium. After that, hairs from their abdominal skin were removed by an electric clip carefully so that the skin was not damaged. Animals were treated according to the principles of care. Approval for these studies was given by the Ethics Committee of Ahvaz Jundishapur University of Medical Sciences (Permit no.: IR.AJUMS.REC.1396.252).^22^

###  Finasteride assay 

 Finasteride amounts were determined using the UV spectroscopy method at λ_max_ = 224 nm.^23^

###  In vitro skin permeation experiment

 Vertical glass Franz diffusion cells (surface area = 4.906 cm^2^) were utilized for skin permeability experiments. Whole skins were completely treated for two and four hours using 2 mL of each of penetration enhancers (Urea, LPGSE, and clove oil) on the surface of the skin at the donor section. finasteride (1% w/v) was dispersed in the distilled water and placed in the donor compartment and the receptor cell, which was filled with phosphate buffer solution (pH 7) and methanol (2:1). The receptor environment is stirred by a small magnetic bead at 300 rpm. At specified intervals (0.5, 1, 2..., 8, and 24 hours), 2 ml of the receptor media was removed and immediately replaced with a fresh phosphate buffer-methanol solution to keep sink conditions. Then, the collected samples were filtered, and finasteride amounts were determined by UV spectrometry at 224 nm.^24,25^

###  Differential scanning calorimeter (DSC)

 To investigate the changes in the skin structure after the pretreatment via penetration enhancers, the DSC technique was employed. Full skin samples were first treated with 2 mL of each penetration enhancers for a period of two and four hours, respectively. Approximately 6-10 mg of skin samples were placed into the sealed aluminum pans. At the same time, a blank pan was used as a reference. The skin samples were exposed to heat ranging from 20 to 200°C (scan speed: 5°C/min). All experiments were completed at least three times. In order to ensure the accuracy and repeatability of the data, the DSC analyzer was calibrated using the indium standard.^26,27^

###  FT-IR spectroscopy

 Skin samples were treated using clove oil, urea, or LPGSE for two and four hours. The samples were then vacuum-dried (650 mm Hg, 25°C) for one hour and finally stored in desiccators to remove traces of the solvent. The skin samples were analyzed in a 4000 to 500 cm ^-1^ scan range using an FT-IR apparatus (Uker, Vertex70, Germany).^28^

###  Statistics and data analysis

 The steady-state flux (mg/cm^2^/h) was calculated from the linear section of the slope of the penetration curve.

 The permeability coefficient factor (K_p_, cm/h) of finasteride by the skin was calculated as follows (equation 1).^29^


$$Eq1\to K_{p}=\frac{J_{ss}}{C_{v}}$$

Where J_ss_ is the rate of the steady-state (flux) and C_v_ is the initial concentration of finasteride. The enhancement ratio (E_R_) is the permeability parameter after the skin had been treated with penetration enhancers divided by the same parameters for non-treated skin (control) (equation 2).^25^


$$Eq2\to ER=\frac{permeability\;parameter\;after\;treatment}{permeability\;parameter\;beforetreatment}$$

Diffusivity was calculated using equation 3.^30^


$$Eq3\to D=\frac{h^{2}}{6Tlag}$$

Where, h is the thickness of the skin, and the lag time (Tlag) parameter was calculated by extrapolating the line of steady-state onto the time axis.

 Statistical analysis was performed using a one-way ANOVA, and *P* < 0.05 was considered significant.^31^

## Results and Discussion

###  Effect of penetration enhancers on the skin permeation of finasteride

 The permeability parameters after skin exposure with penetration enhancers are shown in Table 1. All enhancers affected the flux to a greater extent than diffusivity. The results indicate that all enhancers increased permeability through the skin significantly when compared with control. Two and four hours of pretreatment using clove oil increased the flux of finasteride by 10.09 and 14.35 times, respectively. These results indicate that the time of exposure to clove oil can improve the penetration of finasteride into the skin.

**Table 1:** Permeability parameters after pretreatment with penetration enhancers (Mean ± SD, n = 3)

**Enhancer**		**Parameter**						
		**Flux (mg.cm** ^-2^**.h** ^-1^**)**	**D (cm** ^2^ **.h** ^-1^**)**	**T**_lag_ ** (h)**	**P(cm/h)**	**ER**_flux_	**ER**_D_	**ER**_P_
Control (water)		0.0092 ± 0.0001	0.0036 ± 0.0003	13.956 ± 0.21	0.000920 ± 0.00001	-	-	-
Clove oil	2 h	0.0928 ± 0.10	0.0189 ± 0.0227	9.252 ± 11.047	0.00928 ± 0.01	10.09 ± 1.874	5.254 ± 0.2	10.09 ± 1.8
	4 h	0.132 ± 0.002	0.0237 ± 0.001	2.125 ± 0.1056	0.0132 ± 0.0002	14.35 ± 0.2	6.57 ± 0.3	14.35 ± 0.2
Urea	2 h	0.0231 ± 0.01	0.014 ± 0.01	5.78 ± 0.33	0.0023 ± 0.001	2.51 ± 1.4	31.5 ± 3.3	2.518 ± 1.4
	4 h	0.0200 ± 0.01	0.1138 ± 0.15	4.58 ± 0.8	0.002 ± 0.001	2.18 ± 1.2	4.014 ± 2.7	2.181 ± 1.2
LPGSE	2 h	0.0243 ± 0.008	0.048 ± 0.005	1.05 ± 0.1	0.0024 ± 0.0001	2.647 ± 0.8	13.291 ± 1.409	2.647 ± 0.8
	4 h	0.0546 ± 0.02	0.026 ± 0.01	2.17 ± 1.01	0.0055 ± 0.002	5.935 ± 0.69	7.21 ± 3.3	5.936 ± 0.6

ER_flux_, ratio of flux after and before pretreatment with enhancer; ER_D_ = ratio of diffusion coefficient after and before pretreatment with enhancer.

 Two and four hours of pretreatment with urea increased the flux of finasteride by 2.51 and 2.18 times, respectively. These results indicate that time of exposure to urea can decrease the penetration of finasteride into the skin. Thus, it seems that the effects of the penetration enhancer of urea are reversible after two hours.

 Two and four hours of pretreatment with LPGSEs (1% solution), which has been used for the first time to enhance penetration in the present study, increased the flux of finasteride by 2.647 and 5.935 times, respectively.

 T_Lag_ refers to the limited time that a drug appears in the systemic circulation following extravascular administration. This parameter reflects the processes associated with the adsorption phase from the drug delivery system and the migration of the adsorption surface. Failure to determine T_Lag_ can lead to incorrect estimations of pharmacokinetic parameters.^32^ The greatest reduction in T_Lag_ is related to two-hour LPGSEs, and so this uptake can greatly reduce the duration of transdermal drug delivery. The highest drug diffusivity (ER_D_ = 31.5) was recorded when urea was used for two hours.

###  Differential scanning calorimetry 

 The thermal behavior of various penetration enhancers that were used to pretreat the skin was evaluated based on mean phase transition temperature (Tm) and enthalpy (ΔH). The displacement of the phase transition temperature to lower temperatures indicates a lipid bilayer disorder and an irreversible denaturation of the protein structure in the stratum corneum. Meanwhile, the decrease in the ΔH indicates lipid fluidization in the lipid bilayer and lipid-protein complex in the stratum corneum.^33^ In previous studies, Tm_1_ was attributed to the temperature of the lipid transfer from lamellar to irregular state. Tm_2_ represents the melting point of the keratein-lipid complex or the disturbance in the polypeptide groups of lipids, and Tm_3_ is the discontinuous denaturation temperature of the protein in the stratum corneum.^34^

 The amount of mean phase transition temperature and the enthalpy value after the skin was pretreated with penetration enhancers are shown in Table 2. In previous studies, Vaddi et al reported three endothermic phase transition temperatures for skin (i.e., 62, 79, and 95°C),^28^ while Shakeel et al observed four changes in the endothermic phase transition temperatures (i.e., 34, 82, 105, and 114°C).^35^ Kaushik et al established three endothermic phase transition temperature ranges for stratum corneum (i.e., 59-63°C (Tm_1_), 75-82°C (Tm_2_), and 99.5-120°C (Tm_3_). Tm3 occurred throughout the irreversible denaturation of proteins in SC.^36^

**Table 2:** Effect of penetration enhancer on the thermal properties of hydrated rat skin (mean ± SD, n = 3)

**Penetration Enhancer**	**Transition temperature (°C)**		**Transition enthalpy (mJ/mg)**	
	**Tm**_1_	**Tm**_2_	**ΔH**_1_	**ΔH**_2_
Control (water)	67.5 ± 2.1	112 ± 6.6	7.01 ± 0.4	555.1 ± 19.5
Clove oil (4 h)	37 ± 1.2	116 ± 1.0	1.01 ± 0.2	2.17 ± 0.5
Urea (4 h)	37 ± 0.6	89 ± 2.2	2.4 ± 0.5	3.86 ± 0.1
LPGSE (4 h)	37 ± 0.8	138 ± 1.6	2.22 ± 0.2	45.83 ± 3.1

Tm_1_ = mean transition temperature of lipids; SC Tm_2_ = mean transition temperature of irreversible denaturation of intracellular stratum corneum keratin; ΔH_1_ = transition enthalpy of lipid phase SC ΔH_2_ = transition enthalpy of keratin phase SC.

**Figure 1:**
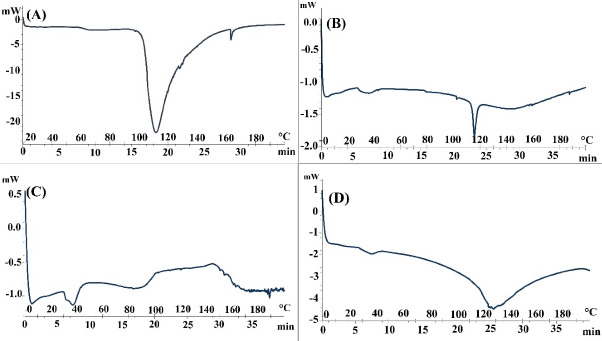
DSC Thermogram of whole skin abdominal rat after pretreatment with (A): Water, (B): Clove oil, (C): Urea and (D): LPGSE.

 DSC thermograms of skin samples pretreated by water and penetration enhancers are shown in Figure 1. In this study, two intraperitoneal transitions of DSC thermograms from hydrated rat skin (about 67°C (Tm_1_) and 112°C (Tm_2_)) were observed. The transmission of Tm_1_ and Tm_2_ seems to be due to the melting of lipids and the irreversible discontinuity of intracellular keratin or melting of lipid (keratin protein) protein. Changes in Tm to lower temperatures can be seen as a two-layer lipid insufficiency and denaturation of proteins in the stratum corneum skin layer, while a decrease in ΔH is related to lipid fluidization in the lipid layer.^28,35,37-39^

 The results of the DSC diagram pretreatment skin by clove oil (2 and 4 hours) indicate decreased Tm_2_ and ΔH_2_ levels. These decreases reflect the melting of the keratin-lipid complex or disorder in the head polar lipids. They also indicate lipid fluidization in lipid bilayers and lipid-protein complexes in the stratum corneum.

 The results of the DSC thermogram of rat skin pretreated with urea (4 hours) suggests that lipid structure is well-regulated in the lipid bilayer. However, a significant decrease in the amount of ΔH_1_indicates the fluidization of the lipid-protein complex in the stratum corneum. The results of the DSC of pretreatment rat skin with urea (4 hours) are largely consistent with the FT-IR results for urea (4 hours). Urea also increases permeability by facilitating the hydration of the stratum corneum and forming hydrophilic channels in this layer. The urea enhancement mechanism involves both hydrophilic activity and lipid disorders in the stratum corneum.^40^

 DSC results from skin pretreated with LPGSE showed an increase in Tm_1_ and Tm_2_ when compared to the control conditions. The results also showed significant increases in ΔH_1_ and ΔH_2_, which do not indicate a disorder in the lipid layer of the skin and the lipid fluidization in the lipid-protein complex in the stratum corneum. These results indicate that LPGSE does not affect the lipid and protein parts of the skin.

###  FT-IR spectroscopy

**Figure 2:**
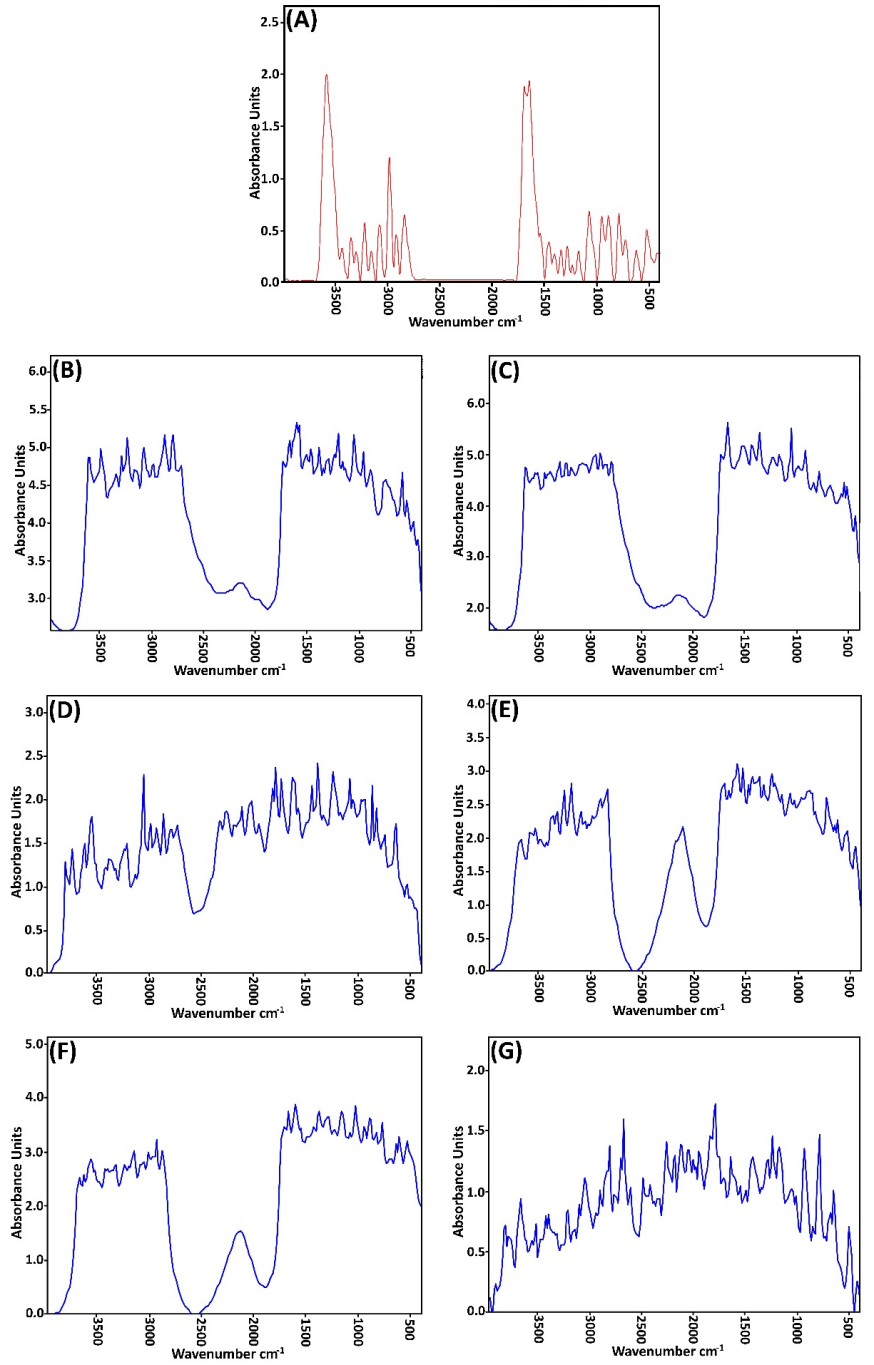
FT-IR spectra of whole skin abdominal rat after pretreatment with (A): Water (B): Clove oil (2 h), (C): Clove oil (4 h), (D): Urea (2 h), (E): Urea (4 h), (F): LPGSE. (2 h), (G): LPGSE (4 h).

Figure 2 and Tables 3-5 show the spectral analysis of the samples concerning any change in the position of the peaks, as well as their severity from the intensity of 3500 cm ^-1^-500 cm ^-1^. Each type of O-H and N-H is found in protein, water, and fat between 3000 and 3600 cm, while the symmetrical and asymmetric stretching bands of the restorative methyl groups of fats are presented at 2948.02 and 2821.65 cm ^-1^. C = O stretching of lipid ester was shown in SC at 1723.62 cm. Also, the stretching of lipid esters in the corneal layer, as well as amide I (stretching C = O) and amide II (stretching C-N) from the secondary spiral structure, was observed in epidermal keratin at 1652.78 and 1572.27 cm ^-1^.^37,41^

**Table 3:** A decrease in mean peak height ( ± SD), compared with control (untreated skin) of asymmetric (Asy) and symmetric (Sym) C-H stretching and C = O stretching absorbance of abdominal hydrated whole skin rat following pretreatment with different vehicles (mean ± SD, n = 3)

**Enhancer**		**Asymmetric C-H stretching**		**Symmetric C-H stretching**		**C=O stretching of lipid ester**	
		**Peak height**	**D%**	**Peak height**	**D%**	**Peak height**	**D%**
Control (Water)		4.877 ± 0.14	-	5.026 ± 0.27	-	4.999 ± 0.17	-
Clove oil	2 h	4.792 ± 0.42	1.743	5.179 ± 0.13	Not Seen	4.837 ± 0.22	3.241
	4 h	5.012 ± 0.36	N.S	4.825 ± 0.11	3.999	5.011 ± 0.27	N.S
Urea	2 h	1.742 ± 0.18	64.281	1.848 ± 0.27	63.231	2.248 ± 0.14	55.031
	4 h	2.587 ± 0.21	46.955	2.754 ± 0.12	45.205	2.823 ± 0.23	43.529
LPGSE	2 h	3.229 ± 0.24	33.791	3.029 ± 0.18	39.733	3.481 ± 0.13	30.366
	4 h	1.125 ± 0.16	76.933	1.009 ± 0.23	79.924	1.315 ± 0.14	73.695

*%Decrease in peak height(%D) = (peak height from untreated whole skin - peak height from solvent treated whole skin)/ peak height from untreated whole skin x 100

**Table 4:** A decrease in mean peak height ( ± SD), compared with control (untreated skin) of C = O stretching (Amide I) and C-N stretching of keratin (Amide II) absorbance of abdominal hydrated whole skin rat following pretreatment with different vehicles (mean ± SD, n = 3)

**Enhancer**		**C=O stretching of keratin**		**C-N stretching of keratin**	
		**Peak height**	**D%**	**Peak height**	**D%**
Control (water)		4.952 ± 0.21	-	4.840 ± 0.28	-
Clove oil	2 h	5.106 ± 0.16	Not Seen	5.336 ± 0.07	Not Seen
	4 h	5.616 ± 0.08	Not Seen	5.184 ± 0.24	Not Seen
Urea	2 h	2.273 ± 0.11	54.099	1.914 ± 0.01	60.455
	4 h	2.787 ± 0.10	43.720	3.124 ± 0.21	35.455
LPGSE	2 h	3.749 ± 0.17	24.293	3.883 ± 0.14	19.773
	4 h	1.287 ± 0.17	74.011	1.052 ± 0.13	78.264

**Table 5:** FT-IR peak wave number (cm ^-1^) changes compared with control (untreated skin) and abdominal hydrated whole skin rat following pretreatment with different vehicles. (mean ± SD, n = 3)

**Enhancer**		**C-H stretching Asy**	**C-H stretching Sym**	**C=O stretching of lipid ester**	**Amide I**	**Amide II**
Control (water)		2948.02 ± 6.04	2821.65 ± 9.95	1723.62 ± 6.52	1651.71 ± 2.63	1572.27 ± 2.41
Clove oil	2 h	2984.24 ± 7.68	2869.90 ± 5.21	1725.06 ± 4.06	1670.56 ± 0.15	1578.88 ± 2.51
	4 h	2952.55 ± 9.51	2846.46 ± 2.51	1725.28 ± 2.59	1669.34 ± 4.52	1516.99 ± 9.87
Urea	2 h	2982.03 ± 5.98	2856.29 ± 8.46	1727.33 ± 9.52	1615.11 ± 2.15	1570.18 ± 5.2
	4 h	2905.02 ± 8.21	2842.60 ± 5.14	1716.91 ± 3.06	1665.41 ± 2.51	1586.29 ± 2.95
LPGSE	2 h	2931.69 ± 5.15	2868.30 ± 7.51	1709.18 ± 5.51	1664.91 ± 3.41	1600.82 ± 0.10
	4 h	3050.28 ± 7.22	2898.79 ± 6.24	1750.25 ± 2.85	1633.36 ± 8.74	1546.08 ± 2.91

 To investigate the mechanism of penetration enhancers’ effects on skin structure by the FT-IR method, shifts in the position of absorbing bands were generally considered to be higher and lower wavelengths and the change in the position of the peaks. If the absorption band is transmitted to higher wavelengths (blue shift), this indicates that the bilayers are fluid in the stratum corneum layer membrane, which impairs the barrier properties and potentially increases the passage of the drug from the skin. On the other hand, a shift of the absorption band towards the lower wavelength (red shift) indicates the reordering of the lipid bilayer groups of the stratum corneum, which ultimately prevents the introduction of drugs into the skin.^42^

 If the penetration enhancer affects the lipid bilayer of the stratum corneum, the phase transition temperature of the lipids increases or decreases in peak wavelengths at 2920 cm ^-1^, 2850 cm ^-1^, and 1738 cm ^-1^. As the height (or intensity) or position of the absorbing bands shows the amount of lipids or proteins in the horny layer, increasing the peak intensity indicates a strengthened lipid structure and causes changes in drug permeability. The decrease in peak intensity, on the other hand, represents a weakening of the lipid structure in the horny layer, which increases the permeability of the drug.^43^

 The FT-IR spectrum of the pretreated urea skin and the decreases in the peak heights of the asymmetric C-H, symmetric C-H, C = O, and amid I, II bands have been created. It seems that the effect of urea enhancement manifests mostly in the lipid and protein content of the stratum corneum and interferes with the stratum corneum tissue keratein. The transfer of absorption bands in symmetric and asymmetric stretching CH regions in the FT-IR spectrum of skin (which was treated by urea to lower wavelengths (red shift)) shows the reordering of bilayer lipid and protein groups.^44^

 Delgado-Charro et al have shown that the use of urea as a penetration enhancer in the solvent system has different effects; however, this is generally the case with lipid disorders in the skin.^45^ Williams and Barry argued that enhancing the permeability of urea, due to increased moisturizing effects, increases the levels of water in the stratum corneum.^17^ Shaikh et al have shown that urea increases permeability by facilitating the hydration of the stratum corneum and by forming the hydrophilic channel channels in this layer. As such, they stated that the urea absorption mechanism involves hydrophilic activity as well as a lipid disorder in the layer stratum corneum.^46^

 In this study, all of the penetration enhancers with different effect times (except for LPGSE (4 h)) transferred the absorption band of non-symmetric C-H to higher wavelengths. This transition is due to the fluidity of the bilayers in the stratum corneum and the impairment of barrier properties, which ultimately increases the passage of medicine into the skin.

 The FT-IR spectrum of clove oil-treated skin indicates that this compound affects the lipid layer of the stratum corneum. The pretreatment time of this compound had a significant effect on the permeability of finasteride on rat skin.

 The FT-IR spectrum of the pretreated skin with LPGSE shows that this combination affects the lipid section and (to some extent) the protein section of the skin.

## Conclusion

 In this study, it was found that all penetration enhancers significantly increased the penetration of finasteride into the skin of the rats. The enhancer effects of clove oil and LPGSE were increased by lengthening the pretreatment time. However, lengthening the pretreatment time did not significantly influence the effect of urea. This result could indicate a reversible increase in urea from increasing the pretreatment time. The results indicate that pretreatment time can increase the permeability of drugs into the skin. Therefore, in order to increase efficacy, the optimal pretreatment time should be determined.

 Two-layer fat disorder and lipid fluidization in two layers of lipids and lipid proteins are the main causes of ER_flux_ and ER_D_. The main barrier to the penetration of finasteride is the skin’s layers. Therefore, a combination of urea, clove oil, and LPGSE can help to achieve suitable skin formulation.

## Acknowledgments

 This paper is derived from the pharm D thesis of one of the authors (Saeed Mohamad Soleymani). Ahvaz Jundishapour University of Medical Sciences is acknowledged for providing financial support.

## Author Contributions

**Conceptualization:** Anayatollah Salimi.

**Data curation:** Saeed Mohammad Soleymani.

**Formal Analysis: **Anayatollah Salimi.

**Funding acquisition: **Hamid Mohammad Soleymani.

**Investigation: **Saeed Mohammad Soleymani.

**Methodology: **Anayatollah Salimi.

**Project administration:** Anayatollah Salimi.

**Resources: **Saeed Mohammad Soleymani.

**Software:** Hamid Mohammad Soleymani.

**Supervision: **Anayatollah Salimi.

**Validation: **Saeed Mohammad Soleymani.

**Visualization:** Hamid Mohammad Soleymani.

**Writing – original draft: **Saeed Mohammad Soleymani.

**Writing – review & editing: **Hamid Mohammad Soleymani.

## Ethical Issues

 Ethical approval was granted by Ethics Committee of Ahvaz Jundishapour University of Medical Sciences (Ethics No. IR.AJUMS.REC.1396.252).

## Conflicts of Interest

 The authors declare no conflict of interest.
